# Letter to the editor: Importance of considering high-risk behaviours in COVID-19 vaccine effectiveness estimates with observational studies

**DOI:** 10.2807/1560-7917.ES.2023.28.4.2300034

**Published:** 2023-01-26

**Authors:** Takeshi Arashiro, Yuzo Arima, Jin Kuramochi, Hirokazu Muraoka, Akihiro Sato, Kumi Chubachi, Kunihiro Oba, Atsushi Yanai, Hiroko Arioka, Yuki Uehara, Genei Ihara, Yasuyuki Kato, Naoki Yanagisawa, Yoshito Nagura, Hideki Yanai, Akihiro Ueda, Akira Numata, Hideaki Kato, Hideaki Oka, Yusuke Nishida, Takao Ooki, Yuki Nidaira, Ashley Stucky, Tadaki Suzuki, Chris Smith, Martin Hibberd, Koya Ariyoshi, Motoi Suzuki

**Affiliations:** 1 Center for Surveillance, Immunization, and Epidemiologic Research, National Institute of Infectious Diseases, Tokyo, Japan; 2 Department of Pathology, National Institute of Infectious Diseases, Tokyo, Japan; 3 Faculty of Infectious and Tropical Diseases, London School of Hygiene and Tropical Medicine, London, United Kingdom; 4 School of Tropical Medicine and Global Health, Nagasaki University, Nagasaki, Japan; 5 Kuramochi Clinic Interpark, Tochigi, Japan; 6 Department of Global Health Promotion, Tokyo Medical and Dental University, Tokyo, Japan; 7 CLINIC FOR Tamachi, Tokyo, Japan; 8 KARADA Internal Medicine Clinic, Tokyo, Japan; 9 Chubachi Internal Respiratory Medicine Clinic, Tokyo, Japan; 10 Department of Pediatrics, Showa General Hospital, Tokyo, Japan; 11 Department of General Internal Medicine, St. Luke’s International Hospital, Tokyo, Japan; 12 Department of Clinical Laboratory, St. Luke’s International Hospital, Tokyo, Japan; 13 Department of Infectious Diseases, Fujita Health University School of Medicine, Aichi, Japan; 14 Machida Ekimae Naika Clinic, Tokyo, Japan; 15 Department of Infectious Diseases, International University of Health and Welfare Narita Hospital, Chiba, Japan; 16 Yanagisawa Clinic, Tokyo, Japan; 17 Shinjuku Home Clinic, Tokyo, Japan; 18 Fukujuji Hospital, Japan Anti-Tuberculosis Association, Kiyose, Japan; 19 Department of Infectious Diseases, Japanese Red Cross Medical Center, Tokyo, Japan; 20 Ikebukuro Metropolitan Clinic, Tokyo, Japan; 21 Infection Prevention and Control Department, Yokohama City University Hospital, Yokohama, Japan; 22 Department of General Internal Medicine and Infectious Diseases, Saitama Medical Center, Saitama, Japan; 23 Saitama Sekishinkai Hospital, Saitama, Japan

**Keywords:** SARS-CoV-2, COVID-19, vaccine effectiveness, behaviour, physical distancing, test-negative design

**To the editor:** We read with interest the article by van Ewijk et al. [1] regarding the influence of people’s behaviour on vaccine effectiveness (VE) estimates against coronavirus disease (COVID-19). We commend the authors’ effort in prospectively collecting detailed exposure history. The authors concluded that it is not necessary to collect data on risk behaviour in a test-negative case–control study, but we believe this conclusion is not fully supported by the data. The VE may be underestimated when there is relaxation of mask/physical distancing policies only among vaccinees or implementation of domestic vaccine certificates/passports to allow vaccinees to engage in high-risk behaviours, as outlined in World Health Organization guidance [2]. In fact, the Netherlands used a ‘coronavirus entry pass’ from 25 September 2021 (midway through the study period), requiring visitors to present the pass at bars, restaurants, events and cultural venues [3]. If the authors had captured this exposure information (i.e. high-risk behaviours associated with this pass), they would probably have seen differing VE estimates with and without adjustments for high-risk behaviours as only the vaccinated would have been allowed to engage in these behaviours. Conversely and counterintuitively, in Table 1, the test-positive group exhibited more frequent mask wearing and more individuals without close contact. The questionnaire could have perhaps asked for more specific exposures such as visiting restaurants/bars, in line with the coronavirus entry pass and previous reports that showed these activities to be high-risk [4,5]. Furthermore, observed waning immunity may partially be due to the introduction of the coronavirus entry pass halfway through the study (i.e. spurious waning), which could have been accounted for with the collection of specific exposures.

There is a previously published report suggesting that policies differentially targeting the vaccinated and unvaccinated would alter VE estimates. A study in New York showed that VE estimates declined simultaneously across different time cohorts after lifting mask mandates exclusively for fully vaccinated individuals, which cannot be explained by waning immunity [6]. Although this potential association was ecological in nature, the study suggested that behavioural changes such as mask wearing may influence VE estimates.

We previously published a similar study adjusting for high-risk behaviours and mask wearing as well as testing behaviour [7]. We also did not see a large difference in COVID-19 VE estimates before and after adjusting for behaviours. This is expected because the Japanese government did not introduce policies differentially targeting the vaccinated and unvaccinated; and our incorporation of high-risk behaviours and mask wearing as covariates strengthened our observational findings. We also did an exploratory secondary analysis to estimate VEs of 2-dose mRNA vaccine recipients among those who did or did not engage in high-risk behaviours (dining at restaurants/bars at night with alcohol consumption in a group was used as a proxy [5]) compared with unvaccinated individuals who did not engage in high-risk behaviours during the BA.1/BA.2-dominant period, assuming a hypothetical scenario of vaccine passport introduction. The resulting VE estimate was significantly lower among vaccinees with high-risk behaviours (36%; 95% confidence interval (CI): 14–53) than among vaccinees with no high-risk behaviours (56%; 95% CI: 41–67; p < 0.001), indicating that VE can be underestimated by 20% via vaccine passport introduction.

When estimating VE, we assume a causal relationship between vaccination and infection/disease [8] and we rely on observational studies as trials are often not ethically possible. Therefore, we need to carefully consider potential confounders and biases in the design and analysis. These potential confounders are not uniform for any disease or context. This notion is becoming increasingly important as infectious diseases are attracting the attention of the public and influencing behaviours, while more observational studies utilise existing data sources, which may not always contain the information necessary for the appropriate analysis.
